# Supplementary material for: Colorimetric Sensing of Lactate in Human Sweat Using Polyaniline Nanoparticles-Based Sensor Platform and Colorimeter
Source: Biosensors (Basel). 2022 Apr 15;12(4):248. doi: 10.3390/bios12040248 (PMC9027737; doi:10.3390/bios12040248)
Supplement: Supplementary file 1 [file biosensors-12-00248-s001.zip › biosensors-1671117-supplementary.pdf]

Supplementary Material

Colorimetric Sensing of Lactate in Human Sweat Using Polyaniline Nanoparticles-Based Sensor Platform and Colorimeter

Hyun Jung Kim [1,2,†], Insu Park [3,4,†], Seung Pil Pack [2,4,5], Gyudo Lee [2,5,*] and Yoochan Hong [1,*]

1 Department of Medical Device, Korea Institute of Machinery and Materials (KIMM), Daegu 42994, Korea; rlaguswnd99@kimm.re.kr
2 Department of Biotechnology and Bioinformatics, Korea University, Sejong 30019, Korea; spack@korea.ac.kr
3 Holonyak Micro and Nanotechnology Laboratory, University of Illinois at Urbana—Champaign, Urbana, IL 61801, USA; insupark@illinois.edu
4 Biological Clock-Based Anti-Aging Convergence Regional Leading Research Center, Korea University, Sejong 30019, Korea
5 Interdisciplinary Graduate Program for Artificial Intelligence Smart Convergence Technology, Korea University, Sejong 30019, Korea
* Correspondence: lkd0807@korea.ac.kr (G.L.); ychong1983@kimm.re.kr (Y.H.)
† These authors contributed equally to this work.

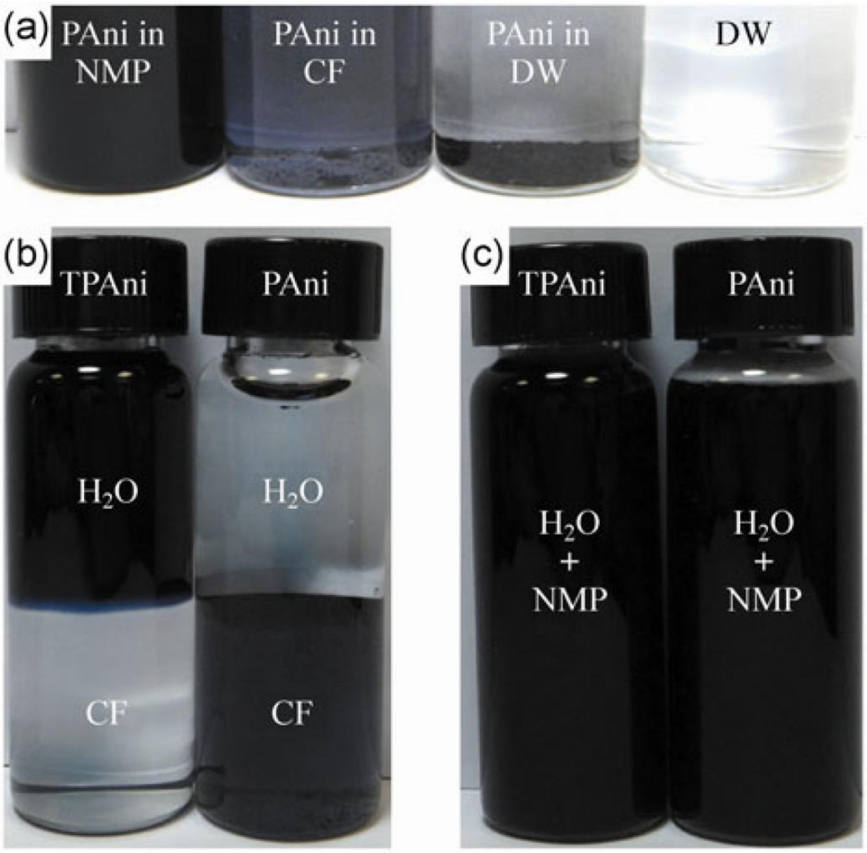

Figure S1. (a) Solubility tests for bare PAni in the various solvents; NMP (N-methyl pyrrolidone), CF (chloroform), and DW (deionized water). (b) Solubility tests for TPAni nanoparticles (0.05 mg/mL) in water and chloroform. (c) Solubility tests for TPAni nanoparticles (0.05 mg/mL) in water and NMP.

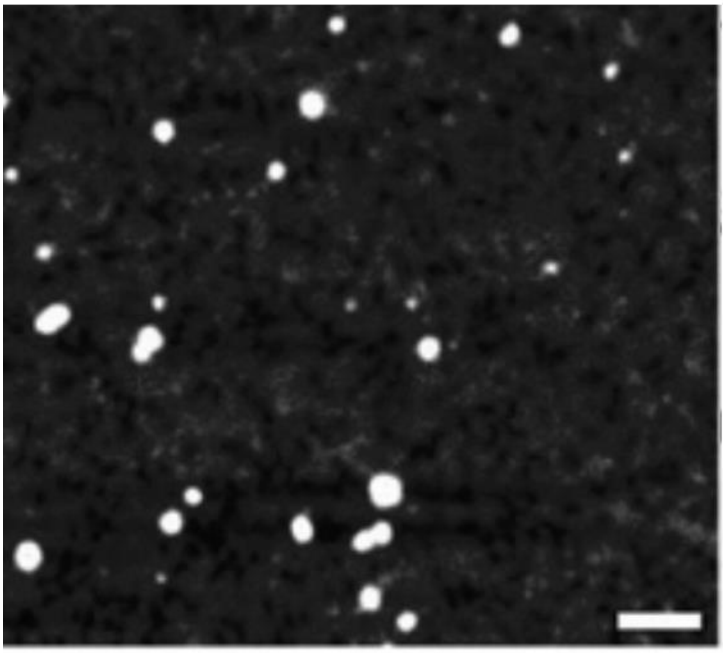

Figure S2. Atomic force microscopic image of the TPAni nanoparticles. The scale bar is 100 nm.

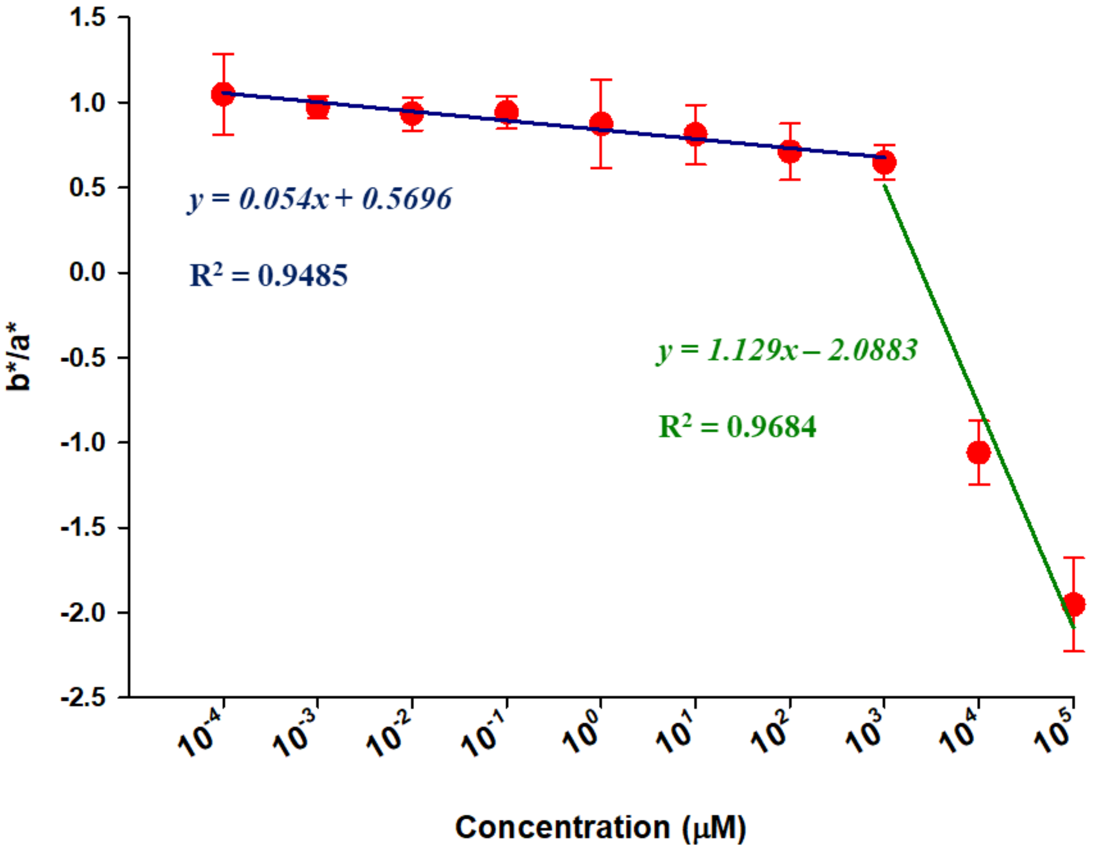

Figure S3. b*/a* values for each concentration of lactate. The fitting lines, its equations, and R2 values for confirming linearity are inserted.
